# Characterization of the Complete Mitochondrial Genome of Three Satyrid Butterfly Species (Satyrinae:Amathusiini) and Reconstructed Phylogeny of Satyrinae

**DOI:** 10.3390/ijms26062609

**Published:** 2025-03-14

**Authors:** Zhicuo Dan, Ying Zhang, Zhenning Chen

**Affiliations:** 1 School of Life Sciences, Qinghai Normal University, Xi’ning 810008, China; danzhicuo@qhnu.edu.cn; 2 Key Laboratory of Zoological Systematics and Evolution, Institute of Zoology, Chinese Academy of Sciences, Beijing 100101, China; zhangying@cnic.cn

**Keywords:** mitochondrial genome, Amathusiini, Satyrinae, phylogeny

## Abstract

Satyrinae, one of the most species-rich groups within the Nymphalidae family, has traditionally relied on morphological characteristics for classification. However, this approach encounters challenges due to issues such as cryptic species and paraphyletic groups. Recent molecular phylogenetic studies have revealed the complex evolutionary history of Satyrinae, leading to the reclassification of the originally polyphyletic Satyrini into multiple independent tribes and confirming the monophyletic status of groups such as Amathusiini. Nevertheless, the phylogenetic relationships and divergence times of certain tribes remain contentious. This study focuses on three species of the Amathusiini tribe (*Faunis aerope*, *Stichophthalma howqua*, and *Aemona lena*), constructing a phylogenetic tree by sequencing the complete mitochondrial genome and integrating 13 protein-coding genes, including COI and ND5. The results indicate that the mitogenome lengths for the three satyrid species are 15,512 bp for *Faunis aerope*, 13,914 bp for *Stichophthalma howqua*, and 15,288 bp for *Aemona lena*. The genetic composition and sequencing of the newly obtained mitogenomes exhibit high conservation and are distinctive to this group of butterflies. Each of the three mitogenomes contains a characteristic collection of 37 genes along with an AT-rich region. Notably, the tRNA genes across these mitogenomes display a conventional cloverleaf configuration; however, the tRNA^Ser^ stem (AGN) lacks the dihydrouridine (DHU) arm. The three species exhibit varying lengths of AT-rich regions, resulting in differences in their mitochondrial genome sizes. Finally, the phylogenetic analysis supports the relationships among the four tribes of Satyrinae as: (Satyrini + (Amathusiini + Elymniini)) + Melanitini.

## 1. Introduction

Satyrinae is the most diverse group of butterflies within the family Nymphalidae (Lepidoptera), comprising approximately 400 genera and 3000 species. This subfamily is found on all continents except Antarctica [1]. Traditionally, Satyrinae is divided into nine tribes and 16 subtribes [2,3]. However, the higher-level classification within this family remains a subject of controversy [2]. Amathusiini, previously classified under Amathusiidae, has been reassessed based on modern research indicating a closer evolutionary relationship with the satyrid lineage [4]. Currently, Amathusiini is recognized as a tribe within Satyrinae. Nonetheless, not all species of Amathusiini are classified within Satyrinae; for instance, *Hyantis* and *Morphopsis* are categorized under Elymniini. Given the complex nature of these relationships, it is essential to utilize mitochondrial genome (mitogenome) sequences for taxonomic and phylogenetic investigations.

The mitochondrial genome of insects is a circular DNA structure, generally measuring between 15,000 and 18,000 base pairs long. It consists of thirty-seven genes, which include thirteen that code for proteins, twenty-two tRNA genes, and two rRNA genes [5]. This genome is essential for evolutionary studies of insects at a systematic level, as its considerable variability and maternal inheritance render it an excellent marker for investigating population genetics and evolutionary connections [5]. In addition, the structural and sequence features of the insect mitochondrial genome provide valuable information regarding mitochondrial functions and adaptive evolution [6,7,8,9,10,11].

In this research, we obtained three new sequences from the Amathusiini tribe. To enhance our understanding of the functions of associated genes, we analyzed the relative synonymous codon usage (RSCU) as well as the AT skew values of protein-coding genes (PCGs), making comparisons with sequences from other Lepidopteran species. Moreover, we constructed a phylogenetic tree for Satyrinae and related groups, investigating the relationships among these taxa. Furthermore, we evaluated the divergence times of three species within the Amathusiini tribe.

## 2. Results

### 2.1. Mitochondrial Genome Assembly and Annotation

**Figure 1:**
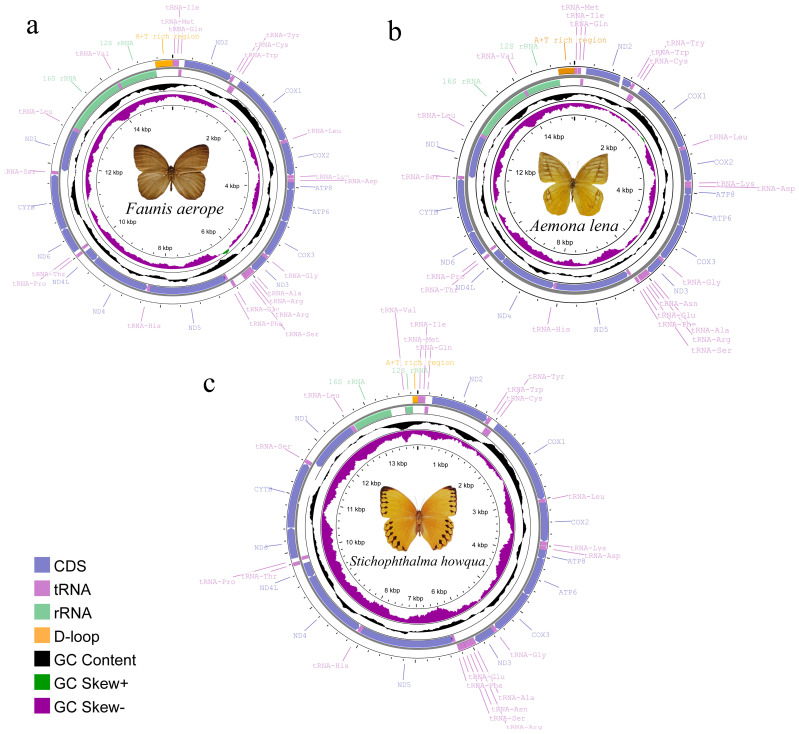
Mitochondrial gene map of three satyrid. (**a**) *Faunis aerope*, (**b**) *Aemona lena*, and (**c**) *Stichophthalma howqua*.

The complete mitogenomes of *Faunis aerope*, *Stichophthalma howqua*, and *Aemona lena* were determined to be 15,512 bp, 13,914 bp, and 15,288 bp in length, respectively. Each genome comprises thirteen protein-coding genes (PCGs), twenty-two tRNA genes, and two rRNA genes, along with an AT rich region (see Figure 1, Table S1). Among the 42 currently published Satyrinae genomes, the mitogenome of *Faunis aerope* is noted as the largest, while *Stichophthalma howqua* also ranks similarly in size. The size of *Aemona lena’s* mitogenome falls within the spectrum of the 42 Satyrinae genomes, ranging from 15,122 bp for *Melanitis leda* to the 15,512 bp of *Faunis aerope* (refer to Table S2). Of the thirty-seven genes encoded by the mitochondrial genome, fourteen are identified as being encoded by the N-strand (the minority strand), which encompasses four PCGs, eight tRNA genes, and two rRNA genes; the remaining twenty-three genes are attributed to the J-strand. Additionally, the AT-rich region was observed to be located between the rrnS and tRNA^Met^ genes.

The A + T content in the mitogenomes of the three satyrid species was 80%, 80%, and 77.9%, respectively, indicating a strong bias toward adenine and thymine over guanine and cytosine. This observation highlights a significant A + T bias within these genomes, as evidenced by the comparison with the G + C content (see Table S2 for detailed data). Moreover, when examining the AT content across various gene components, a consistent decreasing trend emerges. Specifically, the AT-rich region exhibits the highest content, followed by ribosomal RNA (rRNA), transfer RNA (tRNA), and protein-coding genes (PCGs), in that order. In terms of AT skew, the values recorded for the three mitogenomes were −0.050, −0.025, and −0.024, respectively. These negative AT skew values across the PCGs and the first through third codons further corroborate the observation that thymine is more abundant than adenine in these regions. The AT skew metrics for all three mitogenomes are consistent with the broader range found in other Satyrinae species, where values span from −0.055 in *Neope muirheadii* to −0.017 in *Hipparchia autonoe*, as presented in Table S2. These findings suggest a characteristic genetic feature shared among satyrid mitogenomes, emphasizing a notable trend in nucleotide composition.

**Figure 2:**
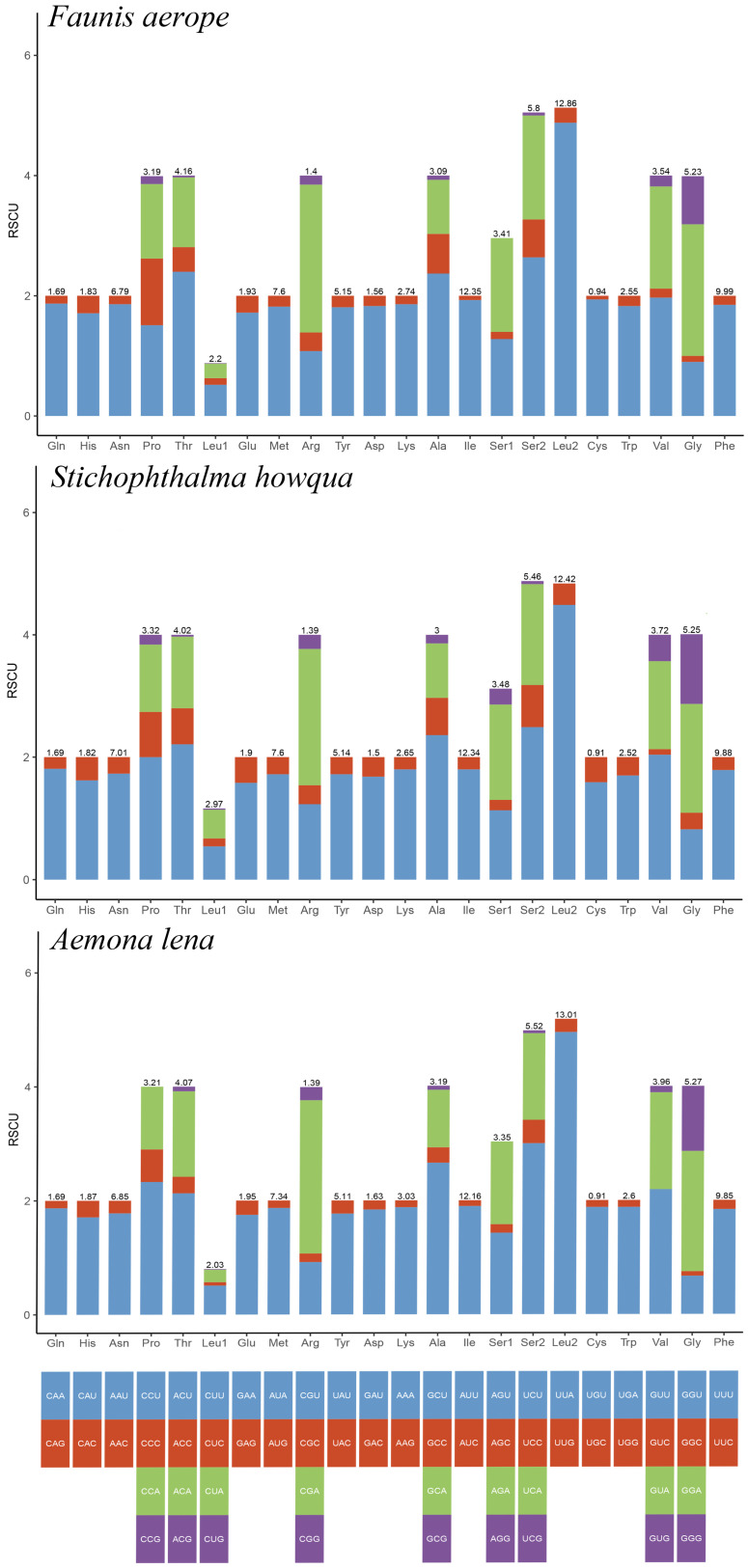
RSCU of *Faunis aerope*, *Stichophthalma howqua*, and *Aemona lena* mitogenome. The bar length represents the accumulated scores of all codons for each amino acid (AA). The numbers on each bar represent the AA ratios of all amino acids in the mitochondral genome.

**Figure 3:**
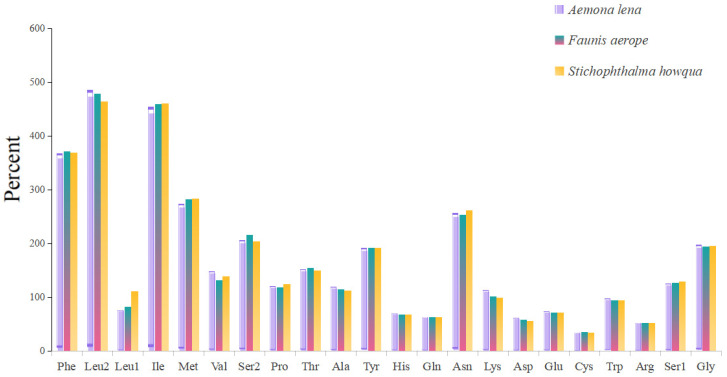
Amino acid composition of protein-coding genes in mitochondrial genome.

The lengths of the 13 protein-coding genes (PCGs) in *Faunis aerope*, *Stichophthalma howqua*, and *Aemona lena* were found to be 11,210 bp, 11,170 bp, and 11,246 bp, respectively. The mitochondrial genomes of these species encode 3736, 3722, and 3748 amino acids (excluding stop codons). This amino acid count falls within the range observed in other Lepidoptera species, aligning closely with counts from 3691 in *Mycalesis intermedia* to 3748 in *Aemona lena* (see Table S2). Figure 2 displays the relative values for codon usage. Notably, AUU (isoleucine; Ile), UUU (phenylalanine; Phe), and UUA (leucine; Leu) were identified as the most commonly utilized codons across the three mitogenomes (Figure 3).

A total of twenty-two standard tRNA genes were discovered in the mitogenomes of three Amathusiini species, with lengths varying between 60 to 72 bp. All identified tRNAs were able to adopt a standard clover-leaf configuration, with the exception of tRNA^Ser^ (AGN), where the dihydrouridine (DHU) arm instead created a loop. The shortened DHU stem observable in tRNA^Ser^ (AGN) is often observed in the mitogenomes of various other insects, including all currently accessible mitogenomes of Satyrinae.

In line with other mitogenomes belonging to the Satyrinae subfamily, the mitogenomes of the three examined species displayed the presence of two ribosomal RNA genes (rrnL and rrnS). These genes were situated between tRNA^Leu^ (CUN) and tRNA^Val^, as well as between tRNA^Val^ and the AT rich region, respectively. The length of the rrnL gene was measured at 1356 bp for *Faunis aerope*, 983 bp for *Stichophthalma howqua*, and 1392 bp for *Aemona lena*. The AT content of the rrnL genes was recorded as 83.8% for *Faunis aerope*, 84.8% for *Stichophthalma howqua*, and 81.4% for *Aemona lena*. The rrnS gene exhibited lengths of 849 bp in *Faunis aerope*, 179 bp in *Stichophthalma howqua*, and 803 bp in *Aemona lena*, with the corresponding AT contents being 84.4%, 92.1%, and 85.8%, respectively. These findings were consistent with those reported for other Satyrinae mitogenomes (Table 1 and Table S2).

### 2.2. Phylogenetic Analyses

**Figure 4:**
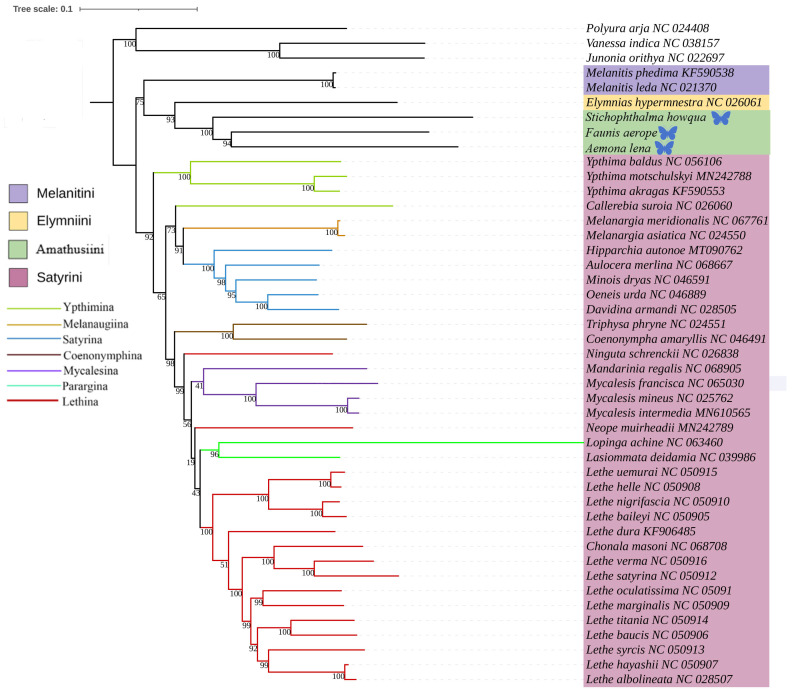
Phylogenetic tree inferred using ML based on nucleotide sequences of thirteen PCG genes and two rRNA genes. Bootstrap supports values expressed in branches. In the phylogenetic tree, species newly sequenced in this study are marked with butterfly-shaped symbols (*Stichophthalma howana*, *Faunis aerope*, *Aemona lena*), while sequence data for all other species were obtained from the NCBI GenBank database.

**Figure 5:**
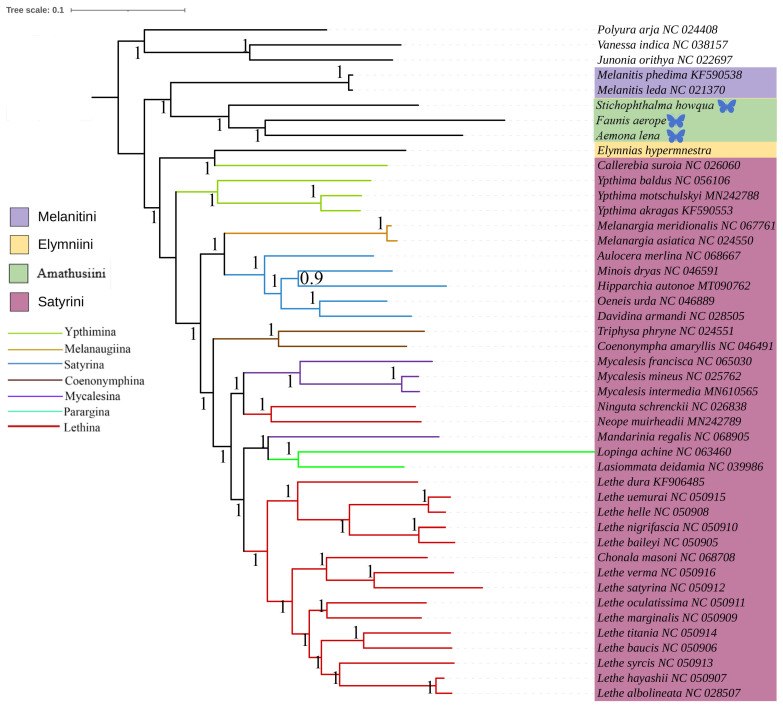
Phylogenetic tree inferred using BI based on nucleotide sequences of thirteen PCG genes and yep rRNA genes. Posterior probabilities are indicated on branches. In the phylogenetic tree, species newly sequenced in this study are marked with butterfly-shaped symbols (*Stichophthalma howana*, *Faunis aerope*, *Aemona lena*), while sequence data for all other species were obtained from the NCBI GenBank database.

This study was based on the aforementioned three newly acquired species of Satyrinae, combined with the known complete mitochondrial genome sequences of 42 other Satyrinae species, whereas the mitogenomes of Nymphalidae (*Junonia orithya*, *Polyura arja*, and *Vanessa indica*) were used as outgroups to reconstruct the phylogenetic relationships of the Satyrinae (see Figure 4 and Figure 5). Both maximum likelihood (ML) and Bayesian inference (BI) methodologies produced consistent topologies concerning relationships at the tribal level. The monophyly of Satyrini received strong support in both ML and BI evaluations.

The phylogenetic relationships among the four tribes of Satyrinae were found to be consistent and are arranged as follows: (Satyrini + (Amathusiini + Elymniini)) + Melanitini. Within the Satyrini, the seven subtribes identified through maximum likelihood (ML) and Bayesian inference (BI) analyses exhibited high similarity, although some differences were noted. The seven subtribes of Satyrini are organized into three branches: *Ypthimina* is positioned alone, while Melanaugiina and Satyrina are grouped together, and Coenonymphina, Mycalesina, Parargina, and Lethina form a separate cluster. At the gene level, *Lasiommata* and *Lopinga* were found to cluster into a single group, subsequently becoming sister taxa to *Lethe*.

### 2.3. Divergence Time Estimation of Satyrinae Species

**Figure 6:**
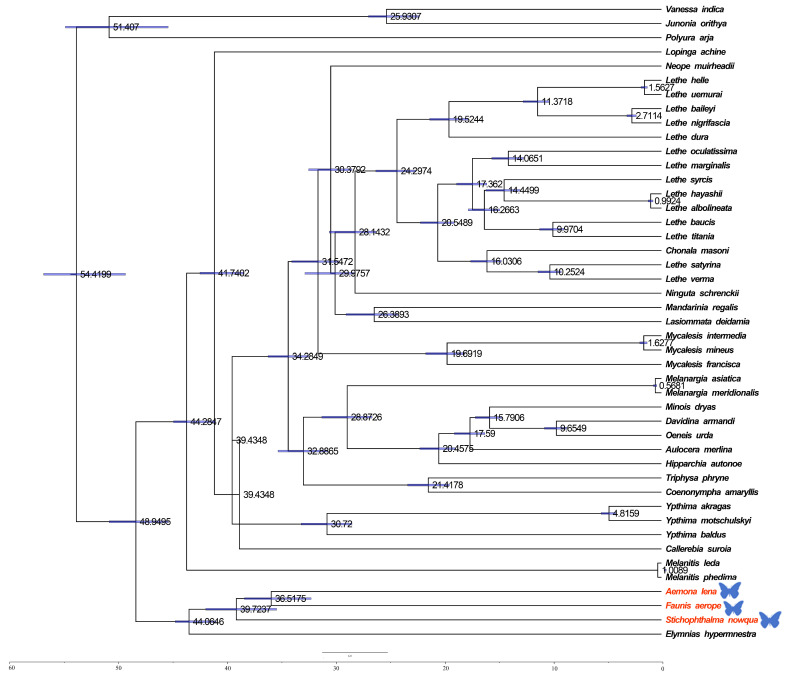
Divergence times for Satyrinae: the maximum clade credibility tree featuring the median age and a 95% confidence interval was estimated through a Bayesian uncorrelated relaxed clock as implemented in BEAST. In the phylogenetic tree, species newly sequenced in this study are marked with butterfly-shaped symbols (*Stichophthalma howana*, *Faunis aerope*, *Aemona lena*).

The estimation of divergence times among Satyrinae was carried out via a Bayesian method applied within BEAST (see Figure 6). The divergence of Satyrinae is dated to the end of the Paleocene in the Paleogene, approximately 48.9495 million years ago (Mya), after which it diverged into two primary branches. One of these clades includes Melanitini, Elymniini, and Amathusiini, with a total divergence time occurring in the Eocene of the Paleogene, approximately 44.0646 Mya. The divergence of Melanitini is estimated to have occurred during the Middle Miocene of the Neogene, around 1.0089 Mya, while the origin of Amathusiini is dated to the Eocene of the Paleogene, approximately 39.7237 Mya. The second major branch, Satyrini, is estimated to have differentiated during the Oligocene of the Paleogene, around 44.2847 Mya.

The results of this study indicate that the Satyrinae subfamily originated at the end of the Paleocene in the Palaeoproterozoic era, followed by the differentiation of various tribes. This group rapidly spread and diversified during the Oligocene epoch, a process significantly influenced by the geological changes and climate variations of that period. The climate of the Paleogene period (66–23 Mya) exhibited a general trend from warm to cold conditions. Early temperatures fluctuated slightly and rose, peaking in the early Eocene epoch (53–33.7 Mya), before transitioning to a cooling phase in the late Eocene epoch. During the middle and late Paleogene, temperatures declined primarily due to an increase in global ice volume, a decrease in sea level, the expansion of grasslands, and the reduction of vegetation coverage, including the area occupied by tropical broad-leaved forests. The findings of this study suggest that the differentiation of the Satyrinae subfamily, as well as the variations within it, were closely associated with geological events and the distribution of host plants during that time.

### 2.4. Mitogenomic Gene Rearrangements

**Figure 7:**
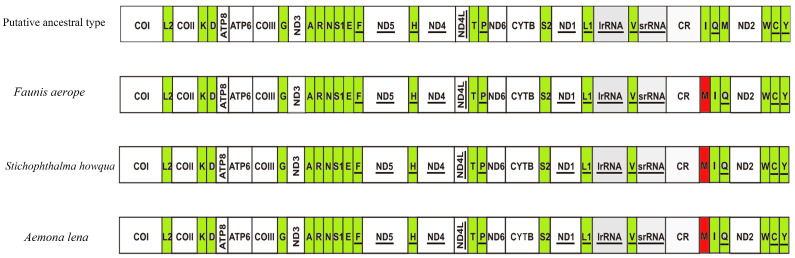
Gene arrangements of *Faunis aerope*, *Aemona lena*, and *Stichophthalma howqua* mitochondrial genomes. The mitochondrial genome is color-coded as follows: white corresponds to protein-coding regions, green designates tRNA genes, and red marks structural rearrangement sites.

The first hexapod to have its mitochondrial genome sequenced was the fruit fly, which exhibits a mitochondrial genome arrangement of trnI-trnQ-trnM. This arrangement is considered the hypothetical ancestral mitochondrial gene arrangement for insects. In the present study, the mitochondrial gene arrangements of the three species of Satyrinae butterflies were found to be trnM-trnI-trnQ, aligning with the gene arrangements observed in other sequenced Satyrinae butterflies (see Figure 7).

## 3. Discussion

### 3.1. Three Satyrid Mitochondrial Genome Structure

The mitochondrial genomes of the three Satyrid species are all typical circular double-stranded structures, with genes highly conserved in terms of length, arrangement order, and nucleotide composition. Among the tRNA secondary structures, except for tRNA^Ser^ (AGN), which lacks the dihydroureoside (DHU) arm and forms a simple loop. The tRNAs form cloverleaf shaped secondary structures, which is consistent with what is seen in other lepidopteran insects. The tRNA^Ser^ (AGN) lacking the DHU arm is a common feature in Lepidoptera mitogenomes [6,7], and this phenomenon has also been found in studies of other insect mitochondrial genomes [8,11]. Mismatches may simply arise from aberrant pairings or be corrected during RNA editing, and it has also been suggested that this particular class of mismatches may increase the efficiency of tRNA work.

In the three Satyrid mitochondrial genomes, a non-standard start codon CGA and a few hexameric start codons were found; the phenomenon of non-standard start codon CGA occurs in the COX1 of most butterflies [9], and not only insects, but the specificity of the mitochondrial genome’s COX1 start codon is prevalent across species of the phylum Arthropoda [10], making COX1 the most variable gene in the arthropod mitochondrial genome; a few hexameric start codons also occur in other butterflies. With the exception of a few genes, the most common termination codon for the three species of satyrid in this study was TAA, while COX2 and ND4 terminated with a single nucleotide, T. In many insects, single nucleotide T as a termination codon is often present in their mitochondrial genomes [11], and during transcription, these single nucleotide termination codons can be used to form a complete termination codon by adding the polyadenylate poly-A at the end again, thus ending the transcription process.

### 3.2. Phylogenetic Analysis of Satyrinae

Among the four tribes of Satyrinae, Satyrini is distinguished by a long branch, consistent with the findings of Peña (2006) [1]. However, the phylogenetic relationships of the remaining three tribes were slightly different from the results derived from previous studies. The phylogenetic relationships in this study consistently resolved their relationship as (Elymniini + Amathusiini) + Melanitini, whereas in other studies, it resolved as (Elymniini + Melanitini) + Amathusiini (ML and BI trees, Wahlberg, 2009) [3]; Elymniini + Melanitini + Amathusiini (MP tree, Wahlberg, 2009) [3]; Melanitini + (Elymniini + Amathusiini) (BI tree, Peña, 2008) [4].

The Satyrinae subfamily comprises various clades, including Elymniini, Amathusiini, Zetherini, Melanitini, and Satyrini [2]. This research reports the determination of three new mitochondrial genomes belonging to Satyrinae, thereby enhancing the total number of available published mitogenomes for this subfamily. Prior investigations have confirmed the close phylogenetic relationships among the Elymniini, Amathusiini, Melanitini, and Satyrini groups, findings that align with our results [4]. However, the subclades within Satyrini exhibited instability across different analyses, indicating the necessity for further molecular sampling and the incorporation of additional genetic markers to establish a more robust phylogenetic relationship for the subfamily.

### 3.3. Mitogenomic Gene Rearrangements in Satyrinae

This study describes a unique rearrangement of the trnM-trnI-trnQ genes in the mitochondrial genomes of the Satyrinae subfamily, which is distinctly different from the arrangement observed in other Hexapoda (trnI-trnQ-trnM). Specifically, trnM has been translocated to a position 5′-upstream of trnI, resulting in the gene order M-I-Q. This rearrangement pattern is highly conserved among three Amathusiini species: *Faunis aerope*, *Stichophthalma howqua*, and *Aemona lena*, and is prevalent in other tribes within Satyrinae [12,13,14,15]. Despite the altered gene order, the tRNAs maintain the standard cloverleaf structure, with the exception of tRNA^Ser^ (AGN), which lacks the DHU arm. This finding indicates that the rearrangement does not significantly impact functionality and may be associated with the high AT bias (with an AT content ranging from 77.9% to 80%) and the mechanisms of replication repair.

## 4. Materials and Methods

### 4.1. Sample Collection and DNA Extraction

Adult specimens of *Faunis aerope*, *Stichophthalma howqua*, and *Aemona lena* were collected in 2021 from Mount Fanjing located in Guizhou Province, China. Initially, these samples were immersed in 100% ethyl alcohol and subsequently stored at −20 °C (School of Life Sciences, Qinghai Normal University, Xi’ning, China). The specimens were identified based on «Chinese Butterfly Fauna (Volumes I and II)» and «BUTTERFLIES OF CHINA (Complete 4 Volumes)» [16,17]. The specimen was photographed using a Nikon D850 (Nikon Corporation, Tokyo, Japan). Utilizing a TIANamp Genomic DNA Kit (Tiangen, Beijing, China), total DNA was extracted from the complete bodies of three specimens, in accordance with the manufacturer’s guidelines. The quality of the DNA extracts was assessed using 1% agarose gel electrophoresis, and the concentrations were determined with a NanoDrop™ 2000 spectrophotometer (Thermo Fisher, Waltham, MA, USA).

### 4.2. Mitogenome Assembly, Annotation, and Analysis

The complete mitogenomes were sequenced using the HiSeq 2500 platform (Illumina, San Diego, CA, USA). This sequencing endeavor was carried out by Biomarker Biotechnologies Inc., located in Beijing, China. The mitochondrial genomes (mitogenomes) of three distinct species were meticulously assembled through the application of the GetOrganell pipeline, version 1.7.5.3 [18]. This robust pipeline facilitated the processing of sequences that were later organized into mitogenomic contigs through the utilization of the Denovo assembly software known as Spades v3.15.5. To predict the thirteen protein-coding genes (PCGs) present within the mitogenomes, a comparative approach was adopted, involving the analysis of homologous sequences derived from reference mitogenomes. This process was complemented by the identification of open reading frames, which was conducted in accordance with the invertebrate mitochondrial genetic code. Furthermore, the positioning of twenty-two transfer RNA (tRNA) genes was accurately identified through the MITOS web server, a tool specifically designed for mitochondrial genome annotation, which was accessed on 20 October 2024 [19]. In addition, the identification of the two ribosomal RNA genes, rrnS and rrnL, along with the AT-rich region, was accomplished by examining the positions of adjacent genes, specifically trnL1 and trnV. This approach was further supported by aligning these sequences with homologous sequences found in reference mitogenomes, ensuring a high level of accuracy in the determination of these genomic elements.

The nucleotide composition, analysis of skew, usage of codons for protein-coding genes (PCGs), and the corresponding values of relative synonymous codon usage for each PCG were assessed using PhyloSuite v1.2.1 [20]. Simultaneously, the tandem repeat units within the AT-control region were examined using the Tandem Repeats Finder online tool (http://tandem.bu.edu/trf/trf.html (accessed on 20 October 2024)) [21]. The shifts in nucleotide composition (composition skew) were determined with the formulas: AT-skew = [(A − T)/(A + T)] and GC-skew = [(G − C)/(G + C)] [22]. Mitochondrial DNA (mtDNA) maps were generated via CGView Server v1.0 [23].

### 4.3. Phylogenetic Analyses and Estimation of Divergence Time

We created a dataset that includes the novel mitogenome of three newly sequenced insect species, along with the complete mitogenomes of 45 additional species obtained from NCBI GenBank (refer to Table 2 and Table S2). The outgroup taxa selected for our analysis were *J. orithya*, *P. arja*, and *V. indica*. To analyze this dataset, we conducted phylogenetic analyses employing both Bayesian inference (BI) and maximum likelihood (ML) methodologies. The alignment of nucleotide sequences was performed using the G-INS-I (accurate) method in codon alignment mode, whereas the amino acid sequences were aligned by employing the auto strategy in normal alignment mode. Furthermore, we leveraged the multiple sequence alignment tool MAFFT available in PhyloSuite v1.2.1 [24] to align nucleotide and amino acid sequences from 13 PCGs present in each mitogenome. The optimal partitioning scheme and nucleotide substitution model for BI and ML phylogenetic analyses with PartitionFinder-2.1.1 were incorporated into PhyloSuite v1.2.1 [25].

In our study, we employed the BEAST v1.8.3 software to estimate divergence times [26]. For the analysis, we utilized a phylogenetic tree constructed from the consensus topology and branch lengths derived from the MrBayes analysis [27]. Three known divergence times were used as calibration points (*Melanitis leda* with *Elymnias hypermnestra*: 57 Mya [28]; *Minois dryas* with *Oeneis urda*: 15.86 Mya [28]; and *Mycalesis intermedia* with *Melanargia asiatica*: 31.5 Mya [29]).

**Table 2:** List of taxa used for phylogenetic analyses in this study.

	Taxon	Mitogenome Size (bp)	GenBank Accession No.	References
	Satyrinae			
	Elymniini			
	*Elymnias hypermnestra*	15,167	KF906484	
	Melanitini			
	*Melanitis phedima*	15,142	KF590538	[30]
	*Melanitis leda*	15,122	JF905446	[14]
	Satyrini			
	*Callerebia suroia*	15,208	NC026060	[31]
	*Coenonympha amaryllis*	15,125	NC046491	[32]
	*Davidina armandi*	15,214	KF881046	
	*Hipparchia autonoe*	15,489	OK094488	[33]
	*Lasiommata deidamia*	15,244	MG880214	[34]
	*Lethe albolineata*	15,248	NC028507	[35]
	*Lethe baileyi*	15,225	NC050905	[7]
	*Lethe baucis*	15,251	NC050906	[7]
	*Lethe dura*	15,259	KF906485	
	*Lethe hayashii*	15,246	NC050907	[7]
	*Lethe helle*	15,253	NC050908	[7]
	*Lethe marginalis*	15,229	NC050909	[7]
	*Lethe nigrifascia*	15,239	NC050910	[7]
	*Lethe oculatissima*	15,243	NC050911	[7]
	*Lethe satyrina*	15,271	NC050912	[7]
	*Lethe syrcis*	15,252	NC050913	[7]
	*Lethe titania*	15,257	NC050914	[7]
	*Lethe uemurai*	15,272	NC050915	[7]
	*Lethe verma*	15,239	NC050916	[7]
	*Minois dryas*	15,194	NC046591	[36]
	*Mycalesis francisca*	15,279	MN242790	[36]
	*Mycalesis intermedia*	15,386	MN610565	[13]
	*Mycalesis mineus*	15,267	KM244676	[37]
	*Neope muirheadii*	15,217	MN242789	[36]
	*Oeneis urda*	15,248	NC046889	[38]
	*Triphysa phryne*	15,143	KF906487	[39]
	*Ypthima akragas*	15,227	KF590553	[30]
	*Ypthima motschulskyi*	15,232	MN242788	[36]
	*Ypthima baldus*	15,304	MN708051	[6]
	*Aulocera merlina*	15,259	NC068667	[40]
	*Lopinga achine*	15,284	NC063460	[41]
	*Mandarinia regalis*	15,267	NC068905	
	*Melanargia asiatica*	15,142	NC024550	[42]
	*Melanargia meridionalis*	15,442	NC067761	
	*Ninguta schrenckii*	15,261	NC026838	[43]
	*Chonala masoni*	15,278	NC068708	
	*Aemona lena*	15,288	This study	
	*Faunis aerope*	15,512	This study	
	*Stichophthalma howqua*	13,914	This study	
Outgroup	*Polyura arja*	15,363	NC024408	[44]
	*Junonia orithya*	15,241	NC022697	[45]
	*Vanessa indica*	15,191	NC038157	[46]

## 5. Conclusions

In this research, we successfully identified three new mitochondrial whole genomes, including *Faunis aerope*, *Stichophthalma howqua*, and *Aemona lena*. Our analysis revealed that their mitochondrial genomes exhibit a remarkable degree of conservation in several key areas. This includes the consistency of base content and composition, the overall size and sequence of the genomes, the presence of protein-coding genes and their corresponding codon usage, as well as the structural characteristics of tRNA secondary structures. To further explore the phylogenetic relationships among these species, we conducted phylogenetic analyses using thirteen protein-coding genes (PCGs) and two ribosomal RNA (rRNA) genes via Bayesian inference (BI) and maximum likelihood (ML) methods. The resulting phylogenetic tree displayed a well-resolved topological structure, with high support for each branch. Our results show that both ML and BI methods produced similar topologies, strongly supporting the identification of well-defined monophyletic groups at the tribe level. These results also elucidate the relationships within the Satyrinae subfamily, specifically supporting the grouping of (Satyrini + (Amathusiini + Elymniini)) + Melanitini. Furthermore, our analysis suggests that the divergence of the Satyrinae subfamily occurred at the end of the Paleocene epoch during the Paleogene period. This temporal insight contributes significantly to the understanding of the evolutionary timeline for these species. Overall, the results of this study offer crucial information that will aid in future investigations regarding the phylogenetic relationships within the Satyrinae subfamily, paving the way for further inquiries and discussions in this area of research.

## Figures and Tables

**Table 1:** Nucleotide composition of *Faunis aerope* (*Fa*), *Aemona lena* (*Ae*), and *Stichophthalma howqua* (*St*).

Feature	Size			A + T%			AT-Skew			GC-Skew		
	*Fa*	*Ae*	*St*	*Fa*	*Ae*	*St*	*Fa*	*Ae*	*St*	*Fa*	*Ae*	*St*
Whole genome	15,512	15,288	13,914	80	80	77.9	−0.050	−0.025	−0.024	−0.224	−0.211	0.304
Protein-coding genes	11,210	11,246	11,170	78.4	78.6	77.7	−0.159	−0.155	−0.161	−0.163	−0.134	−0.217
1st codon position	3736	3748	3722	74.4	73.2	72.1	−0.012	−0.055	−0.026	0.438	0.456	0.464
2nd codon position	3736	3748	3722	70.2	70.3	70.3	−0.36	−0.343	−0.363	−0.194	−0.184	−0.199
3rd codon position	3736	3748	3722	87.8	92.8	90.7	−0.057	−0.107	−0.121	0.7	0.642	0.673
tRNA genes	1445	1450	1415	81	81.2	82	−0.001	0.019	0.007	0.182	0.161	0.157
rRNA genes	849	2195	866	84.3	85.1	80	0.095	0.071	0.062	0.338	0.331	0.260
A + T-rich region	364	345	91	92.1	89.6	75.9	−0.092	−0.043	−0.130	−0.379	0.333	−0.182

## Data Availability

The data are contained within the article or Supplementary Materials.

## Supplementary Materials

The following supporting information can be downloaded at: https://www.mdpi.com/article/10.3390/ijms26062609/s1.
