# Supplementary material for: Characterization of the Complete Mitochondrial Genome of Three Satyrid Butterfly Species (Satyrinae:Amathusiini) and Reconstructed Phylogeny of Satyrinae
Source: Int J Mol Sci. 2025 Mar 14;26(6):2609. doi: 10.3390/ijms26062609 (PMC11942455; doi:10.3390/ijms26062609)
Supplement: Supplementary file 1 [file ijms-26-02609-s001.zip › ijms-3362297-supplementary.pdf]

Table S1 Organization of the three Satyrinae species (*Faunis aerope(Fa)*, *Aemona lena(Ae)*, and *Stichophthalma howqua(St)*) mitochondrial genomes.

Gene	Direction	Location			Size			Intergenic nucleotides			Start/stop codon		
		Fa	*Ae*	*St*	*Fa*	*Ae*	*St*	*Fa*	*Ae*	*St*	*Fa*	*Ae*	*St*
tRN^{Met}	J	1-68	1-66	1-68	68	66	68	0	0	0			
tRNA^{Ile}	J	70-133	73-140	69-132	64	68	64	0	6	0			
tRNA^{Gln}	N	131-199	138-206	130-198	69	69	69	1	-3	-3			
ND2	J	250-1263	252-1265	255-1268	1014	1014	1014	-3	45	56	ATT/TAA	ATT/TAA	ATT/TAA
tRNA^{Trp}	J	1263-1331	1264-1330	1268-1334	69	67	67	50	-2	-1			
tRNA^{Cys}	N	1324-1388	1323-1390	1327-1395	65	68	69	-1	-8	-8			
tRNA^{Tyr}	N	1389-1453	1391-1456	1396-1459	65	66	64	-8	0	0			
COX1	J	1469-3004	1480-3015	1462-2997	1536	1536	1536	0	23	2	CGA/TAA	CGA/TAA	CGA/TAA
tRNA^{Leu}	J	3000-3066	3011-3077	2993-3059	67	67	67	15	-5	-5			
COX2	J	3068-3743	3079-3754	3060-3735	676	676	676	-5	1	0	ATG/T	ATG/T	ATG/T
tRNA^{Lys}	J	3744-3814	3755-3825	3736-3806	71	71	71	1	0	0			
tRNA^{Asp}	J	3824-3890	3828-3892	3812-3881	67	65	70	0	2	5			
ATP8	J	3891-4052	3893-4051	3882-4043	162	159	162	9	0	0	ATC/TAA	ATT/TAA	ATC/TAA
ATP6	J	4046-4723	4045-4722	4037-4717	678	678	678	0	-7	-7	ATG/TAA	ATG/TAA	ATG/TAA
COX3	J	4723-5511	4722-5510	4714-5502	789	789	789	-7	-1	-1	ATG/TAA	ATG/TAA	ATG/TAA
tRNA^{Gly}	J	5514-5578	5513-5578	5505-5569	65	66	65	-1	2	2			
ND3	J	5579-5926	5579-5926	5570-5917	348	348	348	2	0	0	ATT/TAA	ATT/TAA	ATT/TAA
tRNA^{Ala}	J	5928-5993	5928-5591	5926-5991	66	64	66	0	1	8			
tRNA^{Arg}	J	5996-6058	5591-6052	5992-6052	63	62	61	1	-1	0			
tRNA^{Asn}	J	6059-6123	6053-6118	6054-6119	65	66	66	2	0	1			
tRNA^{Ser}	J	6121-6180	6116-6176	6117-6179	60	61	63	0	-3	-3			
tRNA^{Glu}	J	6396-6461	6196-6260	6180-6248	66	65	69	-3	19	0			
tRNA^{Phe}	N	6472-6535	6265-6329	6247-6315	64	65	69	215	4	-2			
ND5	N	6510-8273	6335-8074	6287-8053	1764	1740	1767	10	5	-29	ATT/TAA	ATC/TAA	ATT/TAA
tRNA^{His}	N	8274-8338	8075-8139	8054-8125	65	65	72	-26	0	0			
ND4	N	8339-9677	8140-9478	8126-9464	1339	1339	1339	0	0	0	ATG/T	ATG/T	ATG/T

ND4L	N	9681-9968	9479-9766	9464-9751	288	288	288	0	0	-1	ATG/TAA	ATG/TAA	ATG/TAA
tRNA^{Thr}	J	9971-10034	9769-9832	9754-9818	64	64	65	3	2	2			
tRNA^{Pro}	N	10035-10098	9833-9897	9819-9883	64	65	65	2	0	0			
ND6	J	10101-10631	9900-10430	9886-10419	531	531	534	0	2	2	ATT/TAA	ATT/TAA	ATT/TAA
CYTB	J	10635-11780	10434-11588	10425-11573	1146	1155	1149	2	3	5	ATG/TAA	ATG/TAA	ATG/TAA
tRNA^{Ser}	J	11790-11855	11588-11652	11577-11641	66	65	65	3	-1	3			
ND1	N	11869-12807	11624-12610	11635-12594	939	987	960	9	-29	-7	ATA/TAA	ATA/TAA	ATG/TAA
tRNA^{Leu}	N	12811-12878	12614-12682	12598-12664	68	69	67	13	3	3			
16S rRNA	N	12878-14233	12683-14074	12677-13408	1356	1392	732	3	0	12			
tRNA^{Val}	N	14234-14297	14075-14140	13677-13689	64	66	13	-1	0	268			
12S rRNA	N	14298-15146	14141-14943	13690-13823	849	803	134	0	0	0			
A+T rich		15147-15510	14944-15288	13824-13914	364	345	91						

TableS2 Summarized mitogenomic characteristics of the 42 species investigated in this study

Species	Whole genome			PCGs				16S rRNA		12S rRNA		A+T-rich	
	Size(bp)	A+T(%)	AT-skew	Size(bp)	No.of codons	A+T(%)	AT-skew	Size(bp)	A+T(%)	Size(bp)	A+T(%)	Size(bp)	A+T(%)
Callerebia suroia	15,208	77.5	−0.042	11,195	3730	77.9	−0.166	1347	84.4	753	85.4	417	94.3
Coenonympha amaryllis	15,125	79.5	-0.039	11,228	3741	77.9	-0.166	1337	84.3	759	85.0	308	88.6
Davidina armandi	15,214	79.7	−0.023	11,188	3727	78.2	−0.155	1344	83.9	773	85.0	368	92.9
Elymnias hypermnestra	15,167	80.5	−0.044	11,186	3727	79.1	−0.156	1311	84.7	768	85.0	404	90.6
Hipparchia autonoe	15,435	93.3	−0.017	11,208	3735	76.8	−0.156	1358	83.4	498	83.7	896	94.6
Lasiommata deidamia	15,244	81.1	−0.034	11,191	3729	79.8	−0.156	1321	85.1	762	85.5	417	93.0
Lethe albolineata	15,248	79.2	−0.034	11,188	3727	77.6	−0.160	1339	84.0	772	85.2	413	92.0
Lethe baileyi	15,225	79.0	-0.023	11,208	3735	77.4	-0.155	1345	83.4	769	85.0	410	91.9
Lethe baucis	15,251	78.7	-0.031	11,209	3735	76.7	-0.155	1343	84.2	773	85.5	410	92.9
Lethe dura	15,259	79.2	−0.035	11,233	3743	77.5	−0.169	1341	83.8	752	85.3	432	92.3
Lethe hayashii	15,246	79.0	-0.036	11,206	3734	77.4	-0.160	1337	85.0	763	85.2	416	93.3
Lethe helle	15,253	78.6	-0.019	11,205	3734	76.8	--0.159	1340	84.2	769	85.1	411	91
Lethe marginalis	15,229	80.2	-0.042	11,209	3735	78.6	-0.171	1320	84.5	771	85.9	409	94.4
Lethe nigrifascia	15,239	79.4	-0.023	11,208	3735	77.6	-0.157	1355	83.3	769	85.1	413	92.5
Lethe oculatissima	15,243	79.3	-0.037	11,206	3734	77.4	-0.163	1338	84.6	770	85.1	415	93
Lethe satyrina	15,271	78.8	-0.041	11,209	3735	76.8	-0.165	1338	83.8	769	85	416	93.5
Lethe syrcis	15,252	79.2	-0.038	11,212	3736	77.4	-0.166	1356	84.4	754	84.4	431	93.9
Lethe titania	15,257	78.4	-0.028	11,206	3734	76.7	-0.163	1341	83.0	774	85.0	408	93.1
Lethe uemurai	15,272	78.5	-0.022	11,205	3734	76.6	-0.162	1348	83.6	769	84.9	411	91
Lethe verma	15,239	79.3	-0.042	11,212	3736	77.6	-0.163	1338	83.7	770	86.0	412	93.6
Melanitis leda	15,122	89.5	−0.037	11,194	3730	78.4	−0.166	1332	84.0	774	85.0	314	89.5
Melanitis phedima	15,142	89.7	−0.037	11,179	3725	78.4	−0.166	1329	84.0	780	85.1	183	89.7
Minois dryas	15,194	80.2	−0.028	11,200	3732	78.7	−0.161	1332	84.5	773	85.7	381	94.0
Mycalesis francisca	15,279	79.9	−0.037	11,203	3733	78.1	−0.160	1341	84.9	775	86.2	410	92.7
Mycalesis intermedia	15,386	80.8	-0.029	11,077	3691	79.0	-0.163	1352	85.7	770	85.5	512	93.3
*Mycalesis mineus**	15,267	80.8	-0.030	11,140	3712	79.2	-0.163	1080	84.2	770	85.1		
Neope muirheadii	15,217	80.0	−0.055	11,206	3734	78.5	−0.157	1333	83.9	772	85.9	413	93.0
Oeneis urda	15,248	80.0	-0.022	11,179	3724	78.3	-0.159	1346	84.6	774	85.3	415	94.0
Triphysa phryne	15,143	80.0	−0.036	11,185	3727	77.3	−0.171	1343	84.3	775	84.9	316	83.5
*Ypthima akragas**	15,227	81.9	−0.054	11,206	3734	78.9	−0.160	1326	85.7	546	84.8		
Ypthima motschulskyi	15,232	81.8	−0.051	11,169	3722	80.5	−0.152	1274	85.2	813	86.8	360	93.9
Ypthima baldus	15,304	80.8	-0.045	11,181	3726	80.5	-0.151	1384	84.6	779	85.9	369	91.6
Aulocera merlina	15,295	79.9	-0.031	11,258	3733	79.3	-0.168	1335	84.7	774	85.2	448	92.0
Lopinga achine	15,284	79.4	-0.021	11,274	3735	78.9	-0.160	1333	84.0	775	85.9	450	90.2
Mandarinia regalis	15,267	80.0	-0.025	11,267	3735	79.3	-0.161	1372	85.2	768	84.4	425	92.2
Melanargia asiatica	15,142	79.0	−0.036	11,190	3728	77.5	−0.169	1336	84.4	775	85.6	319	81.9
Melanargia meridionalis	15,138	79.0	-0.033	11,220	3739	77.6	-0.167	1336	84.6	773	86.0	315	81.2
Aemona lena	15,288	80.0	-0.025	11,246	3748	78.6	-0.155	1392	84.8	803	85.8	345	89.6
Ninguta schrenckii	15,261	80.2	−0.053	11,185	3726	78.6	−0.165	1342	84.8	772	84.6	403	92.2

Chonala masoni	15,278	80.7	-0.055	11,302	3737	80.3	-0.165	1340	84.9	771	85.5	414	95.0
Faunis aerope	15,512	80.0	-0.050	11,210	3736	78.4	-0.159	1356	83.8	849	84.4	364	92.1
Stichophthalma howqua	13,914	77.9	-0.024	11,170	3722	77.7	-0.161	983	81.4	179	92.1	91	75.9

Note: * the mitochondrial genome of the indicated species is incomplete.
